# 5,6,7-Trichloro-2-meth­oxy-8-hy­droxy­quinoline

**DOI:** 10.1107/S1600536811010853

**Published:** 2011-04-13

**Authors:** Qiu-Mao Chen, Guo-Bin Yi, Lin-Kun An, Xiao-Long Feng

**Affiliations:** a Faculty of Light Industry and Chemical Engineering, Guangdong University of Technology, Guangzhou 510006, People’s Republic of China; b School of Pharmaceutical Sciences, Sun Yat-Sen University, Guangzhou 510275, People’s Republic of China; c Instrumental Analysis & Research Center, Sun Yat-Sen University, Guangzhou 510275, People’s Republic of China

## Abstract

In the title compound, C_10_H_6_Cl_3_NO_2_, a mean plane fitted through all non-H atoms has an r.m.s. deviation of 0.035 Å. In the crystal, adjacent mol­ecules are connected by O—H⋯O hydrogen bonds and π–π stacking inter­actions [centroid–centroid distance = 3.650 (1) Å], resulting in an infinite chain which propagates in the *b*-axis direction.

## Related literature

The title compound was obtained as an unexpected product from an attempt to synthesize a Top1 (DNA topoisomerase IB) inhibitor For general background to Top1, see: Pommier (2006 ▶). For the synthesis, see: Shen *et al.* (2008 ▶); Cheng *et al.* (2008 ▶).
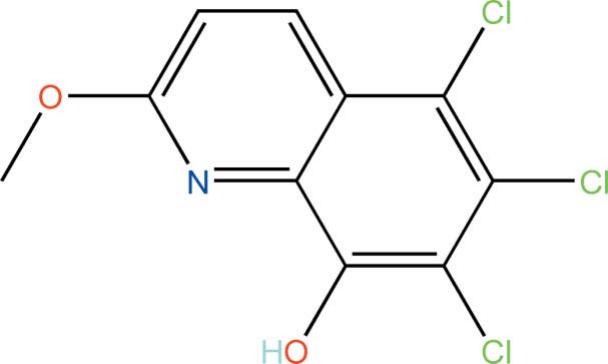


## Experimental

### 

#### Crystal data

C_10_H_6_Cl_3_NO_2_
*M* *_r_* = 278.51
Monoclinic, 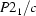

*a* = 10.0782 (3) Å
*b* = 4.9979 (1) Å
*c* = 21.5827 (6) Å
β = 99.287 (2)°
*V* = 1072.87 (5) Å^3^
*Z* = 4
Cu *K*α radiation
μ = 7.61 mm^−1^
*T* = 150 K
0.40 × 0.21 × 0.20 mm

#### Data collection

Oxford Diffraction Xcalibur Onyx Nova diffractometer
Absorption correction: multi-scan (*CrysAlis PRO*; Oxford Diffraction, 2006 ▶) *T*_min_ = 0.151, *T*_max_ = 0.312
4752 measured reflections
2035 independent reflections
1812 reflections with *I* > 2σ(*I*)
*R*_int_ = 0.024

#### Refinement

*R*[*F* ^2^ > 2σ(*F* ^2^)] = 0.039
*wR*(*F* ^2^) = 0.110
*S* = 1.04
2035 reflections
147 parameters
H-atom parameters constrained
Δρ_max_ = 0.98 e Å^−3^
Δρ_min_ = −0.26 e Å^−3^

### 

Data collection: *CrysAlis PRO* (Oxford Diffraction, 2006 ▶); cell refinement: *CrysAlis PRO*; data reduction: *CrysAlis PRO*; program(s) used to solve structure: *SHELXTL* (Sheldrick, 2008 ▶); program(s) used to refine structure: *SHELXTL*; molecular graphics: *SHELXTL*; software used to prepare material for publication: *SHELXTL* and *publCIF* (Westrip, 2010 ▶).

## Supplementary Material

Crystal structure: contains datablocks I, global. DOI: 10.1107/S1600536811010853/nk2089sup1.cif

Structure factors: contains datablocks I. DOI: 10.1107/S1600536811010853/nk2089Isup2.hkl

Additional supplementary materials:  crystallographic information; 3D view; checkCIF report

## Figures and Tables

**Table 1:** Hydrogen-bond geometry (Å, °)

*D*—H⋯*A*	*D*—H	H⋯*A*	*D*⋯*A*	*D*—H⋯*A*
O2—H2*A*⋯O2^i^	0.84	2.25	2.9844 (16)	146

Symmetry code: (i) 
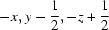
.

**Fig. 1.**
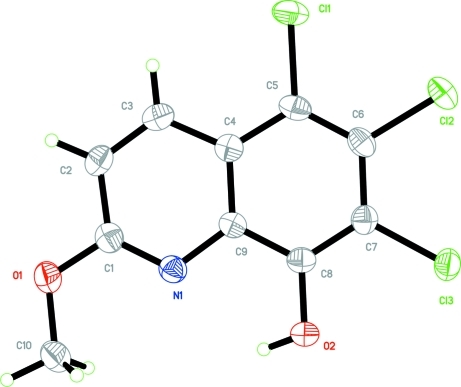
The structure of the title compound, with atom labels and 50% probability displacement ellipsoids for non-H atoms.

**Fig. 2.**
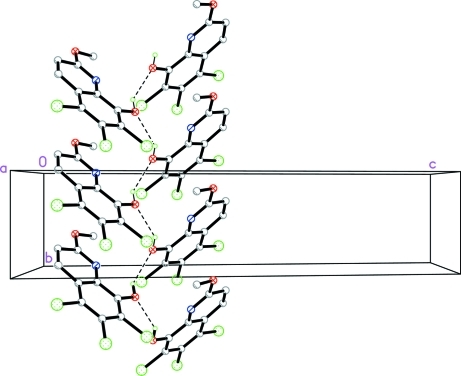
Infinite one-dimensional hydrogen bond in the b-axis direction. C-bound H atoms are omitted.

## supplementary crystallographic information

### Comment

DNA topoisomerase I (Top1) is an essential nuclear enzyme, and can be used as a target to discovery anticancer agents (Pommier, 2006). In our previous effort to find novel Top1 inhibitor, the title compound was obtained as a unexpected product from an attempt to synthesize 6,7-dichloroquinoline-5,8-dione (Cheng *et al.*, 2008 and Shen *et al.*, 2008).

The asymmetric unit of the title compound is shown in Fig. 1. All non-H atoms of the molecule adopt an approximately planar conformation (r.m.s. deviation = 0.035 Å). In the crystal, adjacent molecules are connected by O—H···O hydrogen bonds and π-π stacking interactions [centroid-centroid distance = 3.650 (1) Å], resulting in supramolecular chains along the *b*-axis (Fig. 2).

### Experimental

According to our previously published procedure (Shen *et al.*, 2008), the oxidation of 8-Hydroxyquinoline in concentrated hydrochloric acid with sodium chlorate can give a light yellow solid. The recrystallization of the solid from methanol would give the light yellow crystal.

### Refinement

All H atoms were positioned geometrically and refined using a riding model refined using riding mode. The C—H distances of methyl and benzene ring were 0.98 Å and 0.95 Å, with *U*_iso_(H)=1.5*U*_eq_(C) and 1.2*U*_eq_(C). The O—H distance was 0.84 Å, with *U*_iso_(H)=1.5*U*_eq_(O).

### Crystal data

**Table:**

C_10_H_6_Cl_3_NO_2_	*F*(000) = 560
*M**_r_* = 278.51	*D*_x_ = 1.724 Mg m^−^^3^
Monoclinic, *P*2_1_/*c*	Cu *K*α radiation, λ = 1.54178 Å
Hall symbol: -P 2ybc	Cell parameters from 3109 reflections
*a* = 10.0782 (3) Å	θ = 2.1–71.2°
*b* = 4.9979 (1) Å	µ = 7.61 mm^−^^1^
*c* = 21.5827 (6) Å	*T* = 150 K
β = 99.287 (2)°	Block, light yellow
*V* = 1072.87 (5) Å^3^	0.40 × 0.21 × 0.20 mm
*Z* = 4	

### Data collection

**Table:**

Oxford Diffraction Xcalibur Onyx Nova diffractometer	2035 independent reflections
Radiation source: fine-focus sealed tube	1812 reflections with *I* > 2σ(*I*)
graphite	*R*_int_ = 0.024
Detector resolution: 8.2417 pixels mm^-1^	θ_max_ = 71.4°, θ_min_ = 4.2°
ω scans	*h* = −11→12
Absorption correction: multi-scan (*CrysAlis PRO*; Oxford Diffraction, 2006)	*k* = −6→5
*T*_min_ = 0.151, *T*_max_ = 0.312	*l* = −17→25
4752 measured reflections	

### Refinement

**Table:**

Refinement on *F*^2^	Primary atom site location: structure-invariant direct methods
Least-squares matrix: full	Secondary atom site location: difference Fourier map
*R*[*F*^2^ > 2σ(*F*^2^)] = 0.039	Hydrogen site location: inferred from neighbouring sites
*wR*(*F*^2^) = 0.110	H-atom parameters constrained
*S* = 1.04	*w* = 1/[σ^2^(*F*_o_^2^) + (0.0765*P*)^2^ + 0.4225*P*] where *P* = (*F*_o_^2^ + 2*F*_c_^2^)/3
2035 reflections	(Δ/σ)_max_ = 0.001
147 parameters	Δρ_max_ = 0.98 e Å^−^^3^
0 restraints	Δρ_min_ = −0.26 e Å^−^^3^

### Special details

**Table:**

Geometry. All e.s.d.'s (except the e.s.d. in the dihedral angle between two l.s. planes) are estimated using the full covariance matrix. The cell e.s.d.'s are taken into account individually in the estimation of e.s.d.'s in distances, angles and torsion angles; correlations between e.s.d.'s in cell parameters are only used when they are defined by crystal symmetry. An approximate (isotropic) treatment of cell e.s.d.'s is used for estimating e.s.d.'s involving l.s. planes.
Refinement. Refinement of *F*^2^ against ALL reflections. The weighted *R*-factor *wR* and goodness of fit *S* are based on *F*^2^, conventional *R*-factors *R* are based on *F*, with *F* set to zero for negative *F*^2^. The threshold expression of *F*^2^ > σ(*F*^2^) is used only for calculating *R*-factors(gt) *etc*. and is not relevant to the choice of reflections for refinement. *R*-factors based on *F*^2^ are statistically about twice as large as those based on *F*, and *R*- factors based on ALL data will be even larger.

### Fractional atomic coordinates and isotropic or equivalent isotropic displacement parameters (Å^2^)

**Table:**

	*x*	*y*	*z*	*U*_iso_*/*U*_eq_	
Cl1	0.47126 (6)	0.33323 (15)	0.06402 (3)	0.0429 (2)	
Cl2	0.49339 (5)	0.73083 (11)	0.17798 (3)	0.03287 (19)	
Cl3	0.27565 (6)	0.71889 (11)	0.26624 (3)	0.03218 (19)	
C1	0.0208 (2)	−0.1743 (5)	0.08460 (10)	0.0290 (5)	
C2	0.1158 (2)	−0.1986 (5)	0.04240 (11)	0.0313 (5)	
H2	0.1030	−0.3266	0.0094	0.038*	
C3	0.2240 (2)	−0.0355 (5)	0.05045 (10)	0.0307 (5)	
H3	0.2880	−0.0468	0.0227	0.037*	
C4	0.2422 (2)	0.1530 (4)	0.10032 (10)	0.0263 (5)	
C5	0.3521 (2)	0.3325 (5)	0.11348 (10)	0.0286 (5)	
C6	0.3623 (2)	0.5061 (4)	0.16321 (10)	0.0273 (5)	
C7	0.2632 (2)	0.5050 (4)	0.20283 (10)	0.0262 (4)	
C8	0.1556 (2)	0.3337 (4)	0.19120 (10)	0.0253 (4)	
C9	0.1429 (2)	0.1572 (4)	0.13944 (9)	0.0239 (4)	
C10	−0.1871 (3)	−0.3090 (7)	0.11264 (13)	0.0458 (7)	
H10A	−0.1477	−0.3474	0.1563	0.069*	
H10B	−0.2610	−0.4339	0.0990	0.069*	
H10C	−0.2214	−0.1252	0.1095	0.069*	
N1	0.03253 (18)	−0.0055 (4)	0.13107 (8)	0.0268 (4)	
O1	−0.08538 (18)	−0.3391 (4)	0.07291 (8)	0.0368 (4)	
O2	0.06144 (16)	0.3332 (3)	0.22959 (7)	0.0311 (4)	
H2A	0.0161	0.1917	0.2241	0.047*	

### Atomic displacement parameters (Å^2^)

**Table:**

	*U*^11^	*U*^22^	*U*^33^	*U*^12^	*U*^13^	*U*^23^
Cl1	0.0362 (3)	0.0575 (4)	0.0393 (4)	−0.0108 (3)	0.0186 (3)	−0.0010 (3)
Cl2	0.0250 (3)	0.0306 (3)	0.0418 (3)	−0.0062 (2)	0.0017 (2)	0.0043 (2)
Cl3	0.0324 (3)	0.0297 (3)	0.0335 (3)	−0.0017 (2)	0.0023 (2)	−0.0073 (2)
C1	0.0298 (11)	0.0299 (12)	0.0261 (11)	−0.0015 (9)	0.0009 (9)	0.0037 (9)
C2	0.0351 (13)	0.0340 (12)	0.0242 (10)	0.0016 (10)	0.0029 (9)	−0.0032 (9)
C3	0.0308 (11)	0.0359 (13)	0.0264 (10)	0.0044 (10)	0.0075 (8)	0.0013 (9)
C4	0.0257 (11)	0.0272 (11)	0.0256 (10)	0.0014 (9)	0.0030 (8)	0.0027 (9)
C5	0.0248 (11)	0.0335 (12)	0.0284 (11)	−0.0005 (9)	0.0068 (9)	0.0081 (9)
C6	0.0247 (10)	0.0241 (11)	0.0319 (11)	−0.0018 (9)	0.0013 (8)	0.0057 (9)
C7	0.0277 (10)	0.0220 (10)	0.0284 (10)	0.0023 (8)	0.0027 (8)	0.0007 (8)
C8	0.0245 (10)	0.0262 (11)	0.0257 (10)	0.0024 (9)	0.0057 (8)	0.0030 (8)
C9	0.0229 (10)	0.0244 (11)	0.0242 (10)	0.0007 (8)	0.0035 (8)	0.0036 (8)
C10	0.0373 (14)	0.0620 (18)	0.0399 (14)	−0.0201 (13)	0.0110 (11)	−0.0090 (13)
N1	0.0261 (9)	0.0280 (10)	0.0264 (9)	−0.0019 (8)	0.0047 (7)	0.0016 (7)
O1	0.0368 (9)	0.0393 (10)	0.0346 (9)	−0.0125 (8)	0.0067 (7)	−0.0060 (7)
O2	0.0283 (8)	0.0348 (9)	0.0331 (8)	−0.0051 (7)	0.0132 (7)	−0.0062 (7)

### Geometric parameters (Å, °)

**Table:**

Cl1—C5	1.730 (2)	C5—C6	1.371 (3)
Cl2—C6	1.724 (2)	C6—C7	1.416 (3)
Cl3—C7	1.725 (2)	C7—C8	1.372 (3)
C1—N1	1.302 (3)	C8—O2	1.357 (3)
C1—O1	1.342 (3)	C8—C9	1.413 (3)
C1—C2	1.429 (3)	C9—N1	1.366 (3)
C2—C3	1.350 (3)	C10—O1	1.446 (3)
C2—H2	0.9500	C10—H10A	0.9800
C3—C4	1.420 (3)	C10—H10B	0.9800
C3—H3	0.9500	C10—H10C	0.9800
C4—C9	1.410 (3)	O2—H2A	0.8400
C4—C5	1.418 (3)		
			
N1—C1—O1	120.9 (2)	C8—C7—C6	120.35 (19)
N1—C1—C2	124.0 (2)	C8—C7—Cl3	119.08 (17)
O1—C1—C2	115.1 (2)	C6—C7—Cl3	120.57 (16)
C3—C2—C1	118.5 (2)	O2—C8—C7	119.9 (2)
C3—C2—H2	120.8	O2—C8—C9	119.90 (19)
C1—C2—H2	120.8	C7—C8—C9	120.15 (19)
C2—C3—C4	120.1 (2)	N1—C9—C4	123.60 (19)
C2—C3—H3	120.0	N1—C9—C8	116.37 (18)
C4—C3—H3	120.0	C4—C9—C8	120.0 (2)
C9—C4—C5	118.5 (2)	O1—C10—H10A	109.5
C9—C4—C3	116.5 (2)	O1—C10—H10B	109.5
C5—C4—C3	125.0 (2)	H10A—C10—H10B	109.5
C6—C5—C4	120.9 (2)	O1—C10—H10C	109.5
C6—C5—Cl1	120.67 (18)	H10A—C10—H10C	109.5
C4—C5—Cl1	118.37 (18)	H10B—C10—H10C	109.5
C5—C6—C7	120.0 (2)	C1—N1—C9	117.30 (18)
C5—C6—Cl2	121.00 (17)	C1—O1—C10	116.41 (19)
C7—C6—Cl2	119.02 (16)	C8—O2—H2A	109.5
			
N1—C1—C2—C3	−0.7 (4)	Cl3—C7—C8—O2	−0.4 (3)
O1—C1—C2—C3	178.7 (2)	C6—C7—C8—C9	−0.1 (3)
C1—C2—C3—C4	0.6 (3)	Cl3—C7—C8—C9	179.78 (16)
C2—C3—C4—C9	0.1 (3)	C5—C4—C9—N1	179.7 (2)
C2—C3—C4—C5	179.5 (2)	C3—C4—C9—N1	−0.8 (3)
C9—C4—C5—C6	0.2 (3)	C5—C4—C9—C8	−1.3 (3)
C3—C4—C5—C6	−179.3 (2)	C3—C4—C9—C8	178.2 (2)
C9—C4—C5—Cl1	−178.18 (16)	O2—C8—C9—N1	0.5 (3)
C3—C4—C5—Cl1	2.4 (3)	C7—C8—C9—N1	−179.7 (2)
C4—C5—C6—C7	1.0 (3)	O2—C8—C9—C4	−178.55 (19)
Cl1—C5—C6—C7	179.29 (16)	C7—C8—C9—C4	1.3 (3)
C4—C5—C6—Cl2	−178.17 (17)	O1—C1—N1—C9	−179.3 (2)
Cl1—C5—C6—Cl2	0.2 (3)	C2—C1—N1—C9	0.0 (3)
C5—C6—C7—C8	−1.0 (3)	C4—C9—N1—C1	0.8 (3)
Cl2—C6—C7—C8	178.16 (17)	C8—C9—N1—C1	−178.2 (2)
C5—C6—C7—Cl3	179.09 (17)	N1—C1—O1—C10	2.7 (3)
Cl2—C6—C7—Cl3	−1.8 (2)	C2—C1—O1—C10	−176.6 (2)
C6—C7—C8—O2	179.70 (19)		

### Hydrogen-bond geometry (Å, °)

**Table:**

*D*—H···*A*	*D*—H	H···*A*	*D*···*A*	*D*—H···*A*
O2—H2A···O2^i^	0.84	2.25	2.9844 (16)	146

Symmetry codes: (i) −*x*, *y*−1/2, −*z*+1/2.
